# Comparing Thoracic Epidural Anaesthesia to Rectus Sheath Catheter Analgesia for Postoperative Pain After Major Abdominal Surgeries: A Systematic Review

**DOI:** 10.7759/cureus.48842

**Published:** 2023-11-15

**Authors:** Hussameldin M Nour, Hashim E Elmansi Abdalla, Sameh Abogabal, Abdelwakeel Bakhiet, Abdul Malik Magsi, Muhammad S Sajid

**Affiliations:** 1 Upper Gastrointestinal Surgery, Furness General Hospital, Barrow-In-Furness, GBR; 2 General Surgery, New Cross Hospital, Wolverhampton, GBR; 3 Digestive Disease and General Surgery, Royal Sussex County Hospital, Brighton, GBR; 4 Surgery, Wythenshawe Hospital, Manchester, GBR

**Keywords:** post-operative pain management, post-operative pain, postoperative pain, vas pain score, major open abdominal surgery, laparotomy, rectus sheath catheter, thoracic epidural analgesia

## Abstract

Controlling postoperative pain is essential for the greatest recovery following major abdominal surgery. Thoracic epidural analgesia (TEA) has traditionally been considered the preferred method of providing pain relief after major abdominal surgeries. Thoracic epidural analgesia has a wide range of complications, including residual motor blockade, hypotension, urine retention with the need for urinary catheterisation, tethering to infusion pumps, and occasional failure rates. In recent years, rectus sheath catheter (RSC) analgesia has been gaining popularity.

The purpose of this review is to compare the effectiveness of TEA and RSC in reducing pain following major abdominal surgeries. Four randomised controlled trials (RCTs) reporting outcomes of the visual analogue scale (VAS) pain score were included according to the set criteria. A total of 351 patients undergoing major abdominal surgery were included in this meta-analysis. There were 176 patients in the TEA group and 175 patients in the RSC group. In the random effect model analysis, there was no significant difference in VAS pain score in 24 hours at rest (standardised mean difference (SMD) -0.46; 95% CI -1.21 to 0.29; z=1.20, P=0.23) and movement (SMD -0.64; 95% CI -1.69 to -0.14; z=1.19, P=0.23) between TEA and RSC. Similarly, there was no significant difference in pain score after 48 hours at rest (SMD -0.14; 95% CI -0.36 to 0.08; z=1.29, P=0.20) or movement (SMD -0.69; 95% CI -2.03 to 0.64; z=1.02, P=0.31).

In conclusion, our findings show that there was no significant difference in pain score between TEA and RSC following major abdominal surgery, and we suggest that both approaches can be used effectively according to the choice and expertise available.

## Introduction and background

Pain relief is a basic human right, as per the World Health Organization and the International Association for the Study of Pain [1]. For decades as a profession, we have struggled to manage postoperative pain effectively, with recent studies from the United States of America and Europe failing to show significant improvement in our practice [2]. Laparotomies are operations that are linked to significant degrees of postoperative pain following surgery, mostly because of the extent of the abdominal wall incision [3]. Enhanced recovery protocols have been designed to lower morbidity and help hasten functional recovery, focusing on early mobilisation and early enteral or oral nutrition; this is all to optimise postoperative recovery [4]. Thus, effective analgesia is key to ensuring optimal postoperative recovery by reducing the associated stress response, allowing the patient to mobilise, optimising respiratory function, and helping to avoid gastrointestinal complications such as ileus [5].

There is a wide range of documented complications that can be due to suboptimal pain management, such as increased morbidity, impaired physical function, reduced quality of life, and slowed recovery [6]. Pain has a negative effect on multiple organ systems, including the respiratory system (hypoventilation, decreased vital capacity, pulmonary infection), cardiovascular system (coronary ischemia, myocardial infarction, thromboembolic events), gastrointestinal system (reduced motility, ileus, nausea, vomiting), and renal system (increases in urinary retention and sphincter tone, oliguria). Furthermore, pain can alter immune function, muscular function, and processes such as wound healing. Patients suffering from uncontrolled pain may also experience sleep disturbances, reduced appetite, and psychological effects of pain such as anxiety and becoming demoralised [7]. Lastly, at an organisational level, insufficient pain relief results in prolonged inpatient stays, time to discharge, higher rates of readmission, and an overall increase in the total cost of care [8].

Excessive use of systemic opioid analgesia is associated with a high rate of side effects postoperatively, such as unwanted sedation, compromised respiratory function, pseudo-obstruction or ileus, postoperative nausea and/or vomiting, constipation, urine retention, and, in some cases, itching. Through the use of multimodal analgesics, we can reduce the risk of these effects on our patients [9]. Currently, thoracic epidural analgesia (TEA) is the preferred analgesic modality for major abdominal resections [10]. However, TEA is not without its complications; these include hypotension (with about 20% incidence), residual motor blockade, urine retention, the need for urinary catheterisation, tethering to infusion pumps, and high failure rates [11].

Rectus sheath catheters (RSC) act on structures superficial to the peritoneum and provide pain relief for anterior abdominal wall structures; they are therefore used in procedures requiring a midline incision [12].

Considering this, the aim of this study is to compare the effectiveness of TEA and RSCs in terms of pain relief following major abdominal surgeries.

This article was previously presented as a talking poster at the 2023 Association of Surgeons of Great Britain and Ireland (ASGBI) International Surgical Congress on May 18, 2023.

## Review

Methods

Study Design

This is a systematic review and meta-analysis of comparative studies. This research project, including the scientific writing and submission, is in line with the Preferred Reporting Items for Systematic Reviews and Meta-Analyses (PRISMA) guidelines and the Cochrane Handbook for Systematic Reviews of Interventions [13, 14].

Data Sources and Search Plan

The following electronic databases were searched for randomised controlled trials for this meta-analysis: PubMed, Cochrane Library, Medical Literature Analysis and Retrieval System Online (MEDLINE), and Excerpta Medica database (EMBASE). We utilised Medical Subject Headings (MeSH) search terms to gather purposeful information on randomised controlled trials. The search was broad and conclusive, including multiple aspects, e.g., gender, language, study place of origin, and sample size. We used Boolean operators (AND, OR, NOT) to further widen our results. Articles that were found were studied in depth and assessed against the inclusion criteria. References were also explored as a tool in the selection of suitable trials.

Inclusion Criteria

Papers were required to meet several inclusion criteria to be included in the meta-analysis. Firstly, the study must include a comparison between RCS and TEA following laparotomy surgery. Secondly, the study had to report outcomes of the visual analogue scale (VAS) pain score during rest and mobility states at 24 and 48 hours. Lastly, the papers had to be a randomised controlled trial. No restrictions were made for language, study location, or date of publication.

Data Extraction

Using a pre-established meta-analysis data form, two independent reviewers collaborated to extract pertinent data and reach an agreement. The extracted information on the forms was matched, which resulted in a high and satisfactory inter-reviewer agreement. Data were recorded, including the first author's name, year and country of publication, title of the published study, testing sample size (with gender differentiation if applicable), and the number of patients in each group based on who had TEA or rectus sheath analgesia after midline laparotomy regarding postoperative VAS pain score in 24 and 48 hours. Subsequently, the two reviewers looked at the results, and if any discrepancies were present, the resolution would be through mutual agreement.

Evidence Synthesis

We used the RevMan 5.3 software package (The Cochrane Collaboration, London, UK) for statistical analysis [15]. For the summated outcome of continuous data variables, a standardised mean difference (SMD) with a 95% confidence interval (CI) was used. Furthermore, the random-effects model was used to measure the combined outcomes for both binary and continuous variables [16,17]. Heterogeneity amongst the studies was examined using the chi-squared test, with significance set at p < 0.05, and quantified using the I2 test, with a maximum value of 30% identifying low heterogeneity [18]. We adopted the Mantel-Haenszel method to calculate the relative risk (RR) under the random effect model analysis [19]. As recommended by Deeks et al. [20], we used their method in sensitivity analysis by adding 0.5 to each cell frequency for trials in which no event occurred in either the treatment or control group. In cases where the standard deviation (SD) is not available, we turn to the guidelines provided by the Cochrane Collaboration [16] to calculate it accordingly. For purposes of this method, both groups were stated to have similar variance, which may not have been true, and variance was either assessed from the p-value or the range [21]. The estimate of the difference between both techniques was pooled, depending upon the effect weights in the results determined by each trial estimate variance [21]. A forest plot was created for a graphic illustration of the results; the horizontal line would represent the 95% CI, and the square around the estimate stands for the accuracy of the estimation (sample size). We used the published guidelines of Jaddad et al. and Chalmers et al. [22, 23] to assess the methodological quality of the included trials.

Primary Endpoint

Postoperative pain scores at rest and on movement were analysed after 24 hours as the primary endpoint in this study.

Secondary Endpoint

Postoperative pain scores at rest and on movement were analysed after 48 hours of the laparotomy as the secondary endpoint.

Results

**Figure 1:**
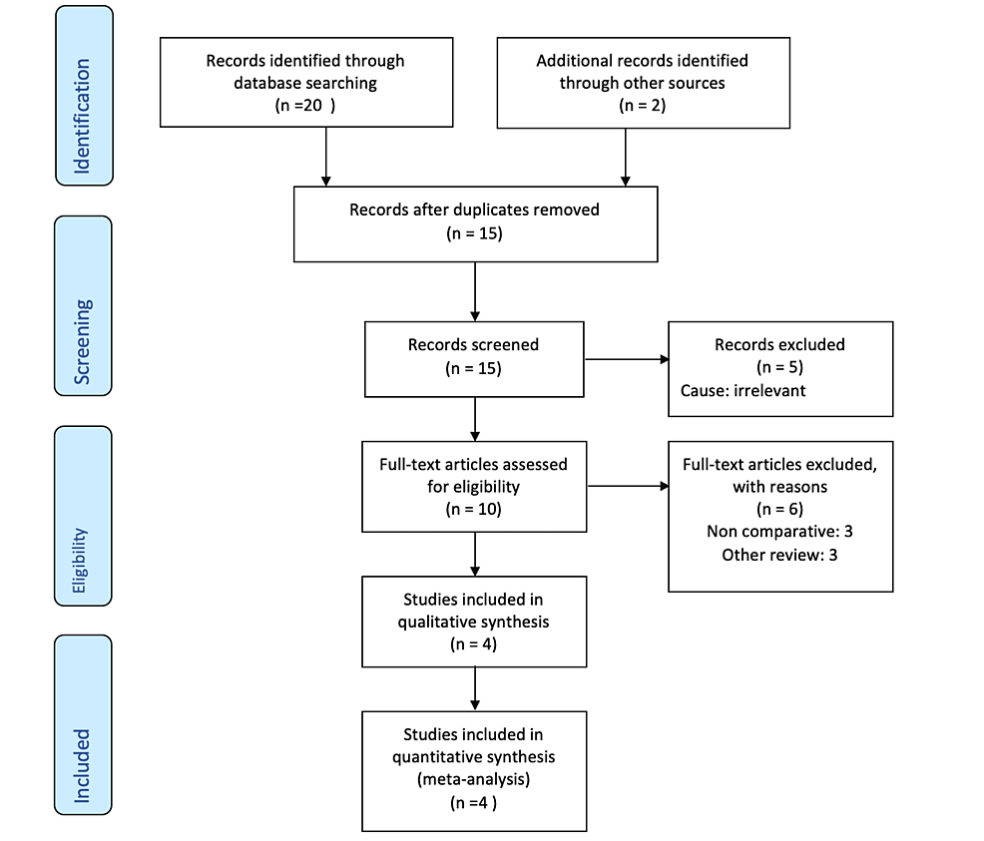
Literature search outcomes illustrated using the PRISMA guidelines PRISMA: Preferred Reporting Items for Systematic Reviews and Meta-Analyses

Our primary search retrieved 22 articles. Seven studies were duplicates, five were found to be irrelevant, three were non-comparative, and three were other reviews. By exclusion, only the four remaining studies were eligible to be included in this review (Figure 1).

Characteristics of Studies and Patients

Four RCTs on 351 patients were included in this meta-analysis [24-27]. Two of the four RCTs were conducted in Egypt and one in India and the United Kingdom, respectively. The number of patients who participated in the trials ranged between 60 and 131. All trials were conducted between 2017 and 2022. The mean age in this review ranged from 47.53 ± 9.43 to 67 years (59-72 years). The main characteristics of the included studies are illustrated in Table 1, and the analgesic technique followed in each of the RCTs is shown in Table 2.

**Table 1:** Characteristics of the included trials TEA: thoracic epidural analgesia; RSC: rectal sheath catheter

Trial	Year	Country	Number of patients	Male to female ratio		Mean age (in years)	
				TEA group	RSC group	TEA group	RSC group
Gupta et al. [24]	2020	India	60	17:13	13:17	56.07 ± 12.94	55.37 ± 12.36
Krige et al. [25]	2022	UK	131	45:20	43:23	67 (59-72)	67 (54-64)
Tueki et al. [26]	2018	Egypt	100	46:4	44:6	53.7 ± 11.6	56.6 ± 8.6
Yassin et al. [27]	2017	Egypt	60	21:10	23:6	48.36 ± 11.73	47.53 ± 9.43

**Table 2:** The analgesic technique adopted in each trial TEA: thoracic epidural analgesia; RSC: rectal sheath catheter

Trial	Type of surgery	TEA group	RSC group
Gupta et al. [24]	Elective open laparotomies	T8 to T10 epidural prior to induction of general anaesthesia (GA). Prior to anaesthetic reversal, a 10 ml bolus of 0.2% ropivacaine was administered epidurally.	Following the laparotomy and prior to extubation, using ultrasound, bilateral rectus sheath block (RSB) catheters were fixed. Then a 20 mL of 0.2% ropivacaine injection was given.
Krige et al. [25]	Elective open laparotomies	Epidural at the level of T7 to T9 for right hemicolectomies and at the level of T9 to T11 for radical cystectomies, left hemicolectomies, and rectal resections. Then a combined bolus of 100 μg fentanyl with 10 ml of 0.25% bupivacaine was given.	Bilaterally inserted via ultrasound, then 20 ml of 0.25% bupivacaine was injected.
Teuki et al. [26]	Elective open laparotomies	Epidural at T9-T10, followed by a bolus dose of 3 mL of lidocaine 2% with 1:200,000 adrenaline, was given, followed by a loading dose of 0.1 mL/kg of 0.125% bupivacaine+fentanyl (2 μg/mL).	Bilaterally inserted via ultrasound, then 20 mL of levobupivacaine (0.25%) and fentanyl (30 μg) were injected.
Yassin et al. [27]	Elective upper abdominal surgery	Epidural at levels T8–T9, then test with 3 mL of lidocaine 1% mixed with epinephrine 1: 200 000. After completion of surgery, analgesia was activated by administering increments of 10 ml of 0.25% bupivacaine over 10 minutes.	Bilaterally inserted via ultrasound, then administration of a 0.25% bupivacaine injection as a 20-ml bolus

Methodological Quality of Included Studies

The methodological quality of the included trials was assessed using the Scottish Intercollegiate Guidelines Network (SIGN) tool [28]. A summary is given in Table 3.

**Table 3:** Quality of the included trials NR: not reported

Trial	Randomization technique	Blinding	Concealment	Intention to treat
Gupta et al. [24]	Via computer-generated random numbers	NR	NR	NR
Krige et al. [25]	Via a computerized system (InForm, version 4.6; Oracle Corporation, Redwood City, CA)	Non-blinded	Computerized system	NR
Teuki et al. [26]	Via computer-generated randomization	Blinded	Via sealed envelops	NR
Yassin et al. [27]	Online randomization program	Blinded	Via sealed envelops	NR

The Mantel-Haenszel random effects model was used to compute robustness and susceptibility to any outlier among these trials. The randomization technique was conducted via computerised systems in all trials. Blinding and concealment via sealed envelopes were reported in two trials [26, 27]. While a computerised system was used in one trial [25], intention to treat analysis was not reported in any of the studies.

Primary Outcomes

**Figure 2:**
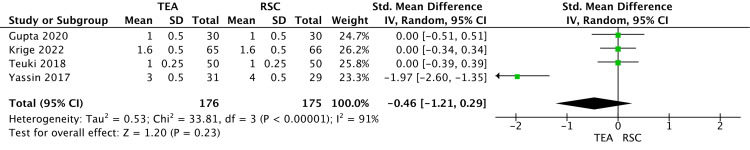
A forest plot shows the VAS pain score following major abdominal surgery at rest after 24 hours. The outcome is presented as the standard mean difference with a 95% confidence interval. [24-27] VAS: visual analogue scale; TEA: thoracic epidural analgesia; RSC: rectal sheath catheter

**Figure 3:**
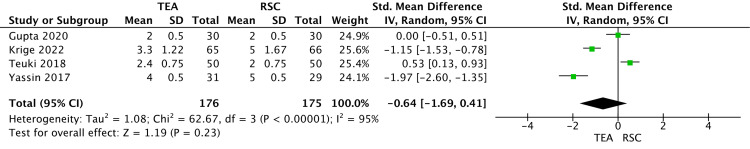
A forest plot shows the VAS pain score following major abdominal surgery on movement after 24 hours. The outcome is presented as the standard mean difference with a 95% confidence interval. [24-27] VAS: visual analogue scale; TEA: thoracic epidural analgesia; RSC: rectal sheath catheter

In the random effect model analysis, there was no significant difference in VAS pain score after 24 hours at rest (SMD -0.46; 95% CI -1.21 to 0.29; z=1.20; P=0.23) (Figure 2) and on movements (SMD -0.64; 95% CI -1.69 to -0.14; z=1.19; P= 0.23) between TEA and RSC (Figure 3).

There was significant heterogeneity among the included trials.

Secondary Outcomes

**Figure 4:**
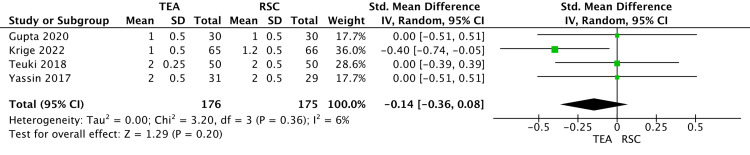
A forest plot shows the VAS pain score following major abdominal surgery at rest after 48 hours. The outcome is presented as the standard mean difference with a 95% confidence interval. [24-27] VAS: visual analogue scale; TEA: thoracic epidural analgesia; RSC: rectal sheath catheter

**Figure 5:**
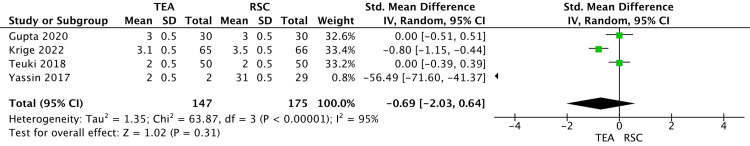
A forest plot shows the VAS pain score following major abdominal surgery on movement after 48 hours. The outcome is presented as the standard mean difference with a 95% confidence interval. [24-27] VAS: visual analogue scale; TEA: thoracic epidural analgesia; RSC: rectal sheath catheter

There was no significant difference in pain score in 48 hours at rest (SMD-0.14; 95% CI -0.36 to 0.08; z=1.29, P=0.20) (Figure 4) and on movements (SMD-0.69; 95% CI -2.03 to 0.64; z=1.02, P=0.31) (Figure 5).

Discussion

This systematic review of four RCTs on 351 patients undergoing open major abdominal surgery has shown that there is no significant difference in VAS pain score between TEA and RSC after 24 hours and 48 hours at both rest and during movement. There is no significant statistical difference between the two groups' pain scores in the random effect model analysis. However, significant heterogeneity was noted, meaning that the included studies are inconsistent due to a reason other than chance.

The present meta-analysis's findings align with those of the previously published meta-analyses on this subject [29]. Due to the lack of RCTs, the previous systematic reviews reported the summated outcome of RCTs alongside retrospective studies, which can generate bias. To our knowledge, this is the first systematic review to include only randomised clinical trials studying the difference in pain scores between TEA and RSC in patients undergoing major abdominal surgery.

This study has several limitations. First, it consists of only four RCTs on 351 patients, which is considered a small number of patients. Moreover, other parameters, such as the need for additional analgesia and the effect on the functional status of the patient's shoulder, must be considered in order to properly assess the effectiveness of any analgesic technique. Furthermore, the safety of these treatment modalities and associated complications should be considered when comparing modes of analgesia. However, this was not possible in this review due to the limited volume of published data. Lastly, there was significant heterogeneity noted amongst the included studies, which can be a potential source of bias.

## Conclusions

Our study concludes that there is no significant difference between TEA and RSC use for pain relief following major abdominal surgeries. This was evident by nearly similar VAS pain scores at 24 hours and 48 hours during both rest and mobility states. We surmise that, according to the literature, both approaches can be effectively utilised as long as the expertise is available.

Due to the limitations of this study, the safety of both methods was not explored. A multicentre RCT with a large study population comparing all elements of a successful mode of analgesia is suggested to determine which mode of analgesia is safer and more effective for postoperative pain relief in major abdominal surgery patients.
